# Context-Aware Healthcare Adaptation Model for COPD Diseases

**DOI:** 10.1007/978-3-030-51517-1_27

**Published:** 2020-05-31

**Authors:** Hamid Mcheick, John Sayegh, Hicham Ajami

**Affiliations:** 8 Digital Research Centre of Sfax, Sfax, Tunisia; 9 Institut Mines-Télécom, CNRS, Paris, France; 10 Université de Sherbrooke, Sherbrooke, QC Canada; 11 Digital Research Centre of Sfax, Sfax, Tunisia; 12 University of Sfax, Sfax, Tunisia; 13 Department of Computer Sciences and Mathematics, University of Québec at Chicoutimi, Chicoutimi, QC G7H 2B1 Canada; 14 Lebanese University, Hadath-I, Beirut, Lebanon

**Keywords:** Software architecture, Self-adaptation, Context-aware system, COPD, Healthcare systems

## Abstract

Nowadays, ubiquitous computing and mobile applications are controlling all our life’s aspects, from social media and entertainment to the very basic needs like commerce, learning, government, and health. These systems have the ability to self-adapt to meet changes in their execution environment and the user’s context. In the healthcare domain, information systems have proven their efficiency, not only by organizing and managing patients’ data and information but also by helping doctors and medical experts in diagnosing disease and taking precluding procedure to avoid serious conditions. In chronic diseases, telemonitoring systems provide a way to monitor the patient’s state and biomarkers within their usual life’s routine. In this article, we are combining the healthcare telemonitoring systems with the context awareness and self-adaptation paradigm to provide a self-adaptive framework architecture for COPD patients.

## Introduction

Chronic obstructive pulmonary diseases (COPD) have attracted research interest as a major public health problem, because according to the World Health Organization (WHO) [1], COPD is currently considered the fourth, and will soon become the third, most frequent cause of death worldwide. It is also a disabling disease and therefore associated with high costs for treating and managing patients. As the disease progresses, patients become more susceptible to respiratory exacerbations which cause frequent hospital admissions and, thus, have a huge impact on patients’ quality of life and healthcare costs [2, 3]. Monitoring patient’s health conditions from home and transmitting these data to a healthcare center could be a great solution that facilitates the management of the growing number of patients with COPD and reduce the burden on health services. This approach is called Home Tele-monitoring, which can be used for a timely assessment of acute exacerbation or as a mechanism to generate alarms to the patients and/or healthcare professionals when clinical changes that may constitute a risk to the patient occur [4]. There are many systematic reviews and studies on the topic of telemonitoring in respiratory patients, specifically in patients with COPD [5–8]. All these studies are focusing on proving the effectiveness of applying home telemonitoring on COPD patients, by studying the services that could be provided and their impacts on the patient’s quality-of-life. However, none of these studies has provided a comprehensive proposal for a telemonitoring system that helps to control the burden of COPD.

We aim to design a telemonitoring healthcare application that helps COPD patients with self-management and improve their quality of life, therefore reducing pressures on healthcare resources. We must develop a system that uses this data to provide an effective intervention that prevents exacerbation through the early recognition of symptoms and prompt treatment which may reduce the risk of hospitalization and control the burden of COPD. Based on this healthcare requirement, we realized the need of combining context awareness and self-adaptation with health telemonitoring, which will give our system the ability to be aware of the patient’s data and context, then to adapt the required changes and act accordingly.

The remainder of this paper is structured as follows. Section 2 introduces the concept of context-awareness and reviews the most common forms of self-adaptation frameworks. Section 3 highlights the characteristics of self-adaptive systems. Section 4 presents our self-adaptation healthcare system for COPD. Section 5 then validates the proposed approach. Finally, Sect. 6 presents our conclusions.

## Context Awareness and Self-adaptation Systems

### Background

The notion of context has appeared implicitly for the first time in the ubiquitous computing area in 1993 by Weiser “all the information that should be taken into consideration for an adjustment” [9]. Lieberman et al. [10] proposed another interpretation of the context that exists within the field of computing: “context can be considered to be everything that affects the computation”. This definition focuses on the application instead of the user, but nowadays with the widespread of mobile applications that focus on the user’s lifestyle, health, and activities the factors that affect the user and these that affect the computation process become almost the same.

In software systems, context awareness notion is mostly coupled with self-adaptation capability otherwise, there is no point in collecting contextual data. Self-adaptation is a set of simultaneous and successive processes as an organized reaction to changes in the resources or environment of the system [11]. Self-adaptive systems dynamically modify their behaviors to respond effectively to changes in their operational environment [12]. In the next section, an overview of many self-adaptation frameworks is provided.

### Self-adaptations Frameworks

Rainbow framework provides general mechanisms for developing reusable self-adaptive systems at a variety of different levels [13]. Model-Driven Approach is an automated self-adaptive model that supports adding and removing technical resources at run-time [14]. Meta-Self is a service-oriented framework that provides a solid platform for the development of SAS [15]. This framework allows designers to identify system properties, architectural patterns, and different adaptation mechanisms. FUSION is a reusable feature architecture that incorporates a learning-based adaptive cycle. The adaptation cycle consists of three main steps—detect, plan, and effect [14]. MOSES is a service-oriented framework that focuses on quality of service (QoS) requirements at runtime. MOSES provides a reusable implementation strategy of the adaptation logic following the MAPE cycle (Monitoring, Analysis, Planning, and Execution) [14]. The Contract-Based Adaptive Software Application framework (CASA) [16] is specialized in handling resources instability. The framework assumes that a system should not make any assumptions about the resources that will be available and should be prepared for any resource availability scenario cases. Service-Oriented Architectures (SSOA) is a software framework that specifies any kind of adaptation by decomposing of functionalities [17]. Each of these functionalities shall be specialized to fit a particular purpose. CareDroid is an adaptation framework for android context-aware applications [18]. This framework CareDroid monitors the contexts at run-time, and active methods only when it intercepts calls to sensitive methods.

## System Requirements and Self-adaptation Characteristics and Taxonomy

### Requirements Extraction and Gathering

The first step to designing a self-adaptive system is to well identify the system requirements; we will depend on W5H-Pattern [19], which presents six questions that would help us in eliciting adaptation requirements (Table 1).

**Table 1.** Requirements extraction

Where	Where do we need to make a change inside our system when a context’s change does happen? Depending on the model presented by Ajami and Mcheick [20], the change needs to be done in the Application layer on both sides: user interface and physician interface
When	When do we need to make these changes? Whenever an urgent update happens in the user contextual data like vital signs, environmental risk factors, and planned activities or periodical changes like the evaluation of treatment and decision support suggestions
What	What do we need to change? We need to update some system attributes that present the system state and these attributes in its turn could trigger new functions or activate new components
Why	Why these changes are required? In healthcare monitoring applications especially these related to chronic diseases, taking precluding actions is crucial in treatment plans. Also being able to notify the patient and the medical experts about any threatening situation or abnormal signs make these kinds of applications more efficient
Who	Is any human intervention is required in the adaptation process? From the patient side, all his biomedical data and surrounding environments data will be collected from sensors. However, because physical activities do affect the COPD patient’s state, he needs to detect his planned physical activity (running, swimming)
How	How to determine what changes and actions are needed to be done in the adaptation process? Ajami and Mcheick [20] provided a rule-based reasoning engine, depending on these generated rules all the required actions and changes can be deduced

### Adaptation Characteristics and Taxonomy

Christian et al. [21] presented a taxonomy of the different properties of self-adaptive software. We will analyze this work and do a projection on our system requirements and use the results to build our system.

#### Time:

Handte et al. [22] provided two perspectives of temporal aspects: (i) Reactive is when we have to adapt whenever a change in the context does happen. (ii) Proactive is when the monitored data is used to forecast system behavior or environmental state [21]. In our case, the adaptation will be reactive depending on the changes that happen in the user contextual data.

#### Reason:

The adaptation could be triggered for three reasons: (i) change of the context, (ii) change in the technical resources, and (iii) change in the users. In our case, the adaptation is triggered due to contextual changes, which provide a potential solution for the multiscale nature of COPD.

#### Level:

In our system, the change needs to be done on the application layer, where we need to update the acceptable range for the different datasets or we need to activate new components or call new functions.

#### Technique:

McKinley [23] provided two techniques for adaptive software: parameter adaptation and compositional adaptation. Parameter adaptation achieves a modified system behavior by adjusting system parameters. Whereas compositional adaptation enables the exchange of algorithms or system components dynamically at runtime. We will use the first approach because it is suitable for a rule-based system.

#### Adaptation Control:

Two approaches for implementing the adaptation logic can be found in the literature. The internal approach, which twists the adaptation logic with the system resources. The external approach splits the system into adaptation logic and managed resources, The IBM Autonomic Computing Initiative provided MAPE Model [24], which is an external, feedback control approach. Another aspect of the adaptation logic is the degree of decentralization. We will follow a decentralized approach by implementing independent units that control different aspects of adaptation.

## Self-adaptation Healthcare System for COPD

### Proposed System

**Fig. 1.**
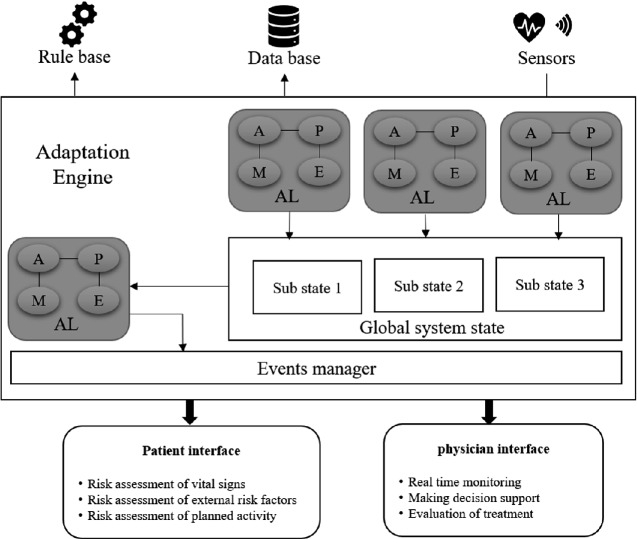
Architecture for COPD context-aware system

Ajami and Mcheick [20] have proposed an ontology-based approach to keep track of the physical status of patients, suggest recommendations and deliver interventions promptly, by developing a decision support system based on an ontological formal description that uses SWRL rules. The main goal of this paper is to provide an adaptation architecture design for the application layer, which will address the connection between three different entities:

- 1 - The end-user application: which is supposed to provide a certain service for both patient and physician.
- 2 - The data sources (sensors and patient’s records): that provides a continuous stream of contextual data and historical data about the patient.
- 3 - The rules base: which presents the knowledge base in our system (Fig. 1)

### The Adaptation Engine and Monitoring Units

The adaptation engine consists of a central adaptation unit and multiple sub adaptation units. Each subunit is responsible for monitoring and managing changes for a specific category tuple (data, rules, services).

The system variables will be saved in a shared memory called the global state, which is a composition of sub-states. Each sub-state is considered as a container for saving category-specific data and it will be updated and managed by the adaptation subunit that is responsible for monitoring the same category. We will divide all our sets of data, rules, and services into three categories (Table 2):

**Table 2.** Categorization of data

	Biometrics	Environmental	Activities
Data	Biometrics data	Environmental data	Activities data
Rules	Biometrics rules	Environmental rules	Activities rules
Services	Biometrics services	Environmental services	Activities services

**Fig. 2.**
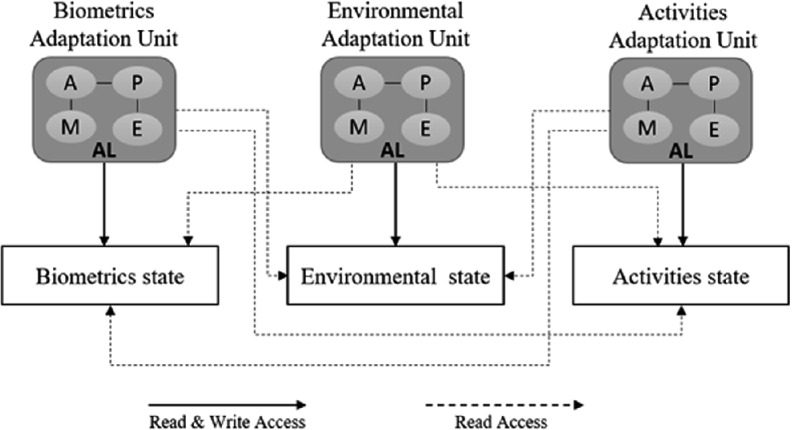
Subunits and sub stats

Now each subunit will be responsible for a specific category of the tuple. Therefore, we will have three subunits: 1 - The biometrics unit 2 - The Environmental unit 3 - The Activities unit (Fig. 2).

- The Biometrics Unit will be responsible for monitoring all the patient’s biomarkers upcoming from the biometrics sensors, and it will read all the stored data in the other two sub-states (the environmental state & the activities state), then depending on the vital related rules in the rules engine, it will update The Biometrics State with the safe ranges for all the vital biomarkers and the current measurements for them. The same workflow will be applied in both the Environmental Unit and the Activities Unit.
- The Central Adaptation Unit: it is considered the main core of our system; it will be responsible for monitoring the global state, which will get rid of the burden of dealing with the continuous streaming of the patient’s biometrics and the environmental data. By collecting all the contextual data in the global state, each category in its sub-state, we will have access to all the current biometrics and external factors value with the safe range for each one of them as well the current physical activity and the planned list of activities.

Depending on the previous data, the central unit will be able to detect any potential risk or abnormal situations by comparing the current value of each factor in the sub-states with its normal range, which had been adapted by every sub adaptation unit.

When an abnormal situation is detected, the central unit will detect what action should be taken to prevent an exacerbation in the patient’s health state.

## Validation

In order to test and validate our proposed system, we implemented a simulation app using some data obtained from medical records to simulate the streamed data and a set of COPD rules extracted in [25] to create some testing scenarios. The main focus of the validation process was on the efficiency of the system to provide continuous monitoring of the patient status, and the ability to apply and adapt the required changes to prevent any dangerous exacerbation. The testing scenarios we had performed, proofed the ability of our system to handle the complexity of monitoring the enormous amount of contextual data, and keep track of the latest updates in the global state. Also, following an aspect-oriented approach facilitates the implementation of the adaptation logic, by separating the categories of data that each Adaptation Unit needs to be responsible for observing. After testing some rules that lead to call a sequential set of actions and multiple updates in the state units, the system was able to adapt the safe ranges for the different environmental and biometrical factors and detect suitable action in an abnormal situation. Nevertheless, our system still needs to be tested when it is connected to the whole rules engine when all COPD rules are inserted into the engine, which will be done in future work.

## Conclusion

In this paper, we have presented an architecture for a context-aware self-adaptive system that is used to develop a COPD healthcare telemonitoring system. The system is backed out by a medical rules engine in the COPD domain that is used as the knowledge base to determine the safe ranges for patient’s biomarkers and external factors, then detect the precluding actions needed to be taken to prevent severe exacerbations in patient’s health state.

Our main contribution in this work is providing a context-aware self-adaptive system architecture that is dealing with the huge variety and complexity of contextual data and different sets of services by implementing a decentralized adaptation unit, which makes the monitoring and adaptation task easier and less complex by applying the separation of concerns principle.
